# Quality in Clinical Consultations: A Cross-Sectional Study

**DOI:** 10.3390/clinpract12040058

**Published:** 2022-07-14

**Authors:** Anneke Graf, Chan Hee Koh, Gordon Caldwell, Joan Grieve, Melissa Tan, Jasmine Hassan, Kaushiki Bakaya, Hani J. Marcus, Stephanie E. Baldeweg

**Affiliations:** 1 Department of Endocrinology, University College London Hospitals NHS Foundation Trust, London NW1 2BU, UK; anneke.graf@nhs.net (A.G.); melissa.tan6@nhs.net (M.T.); jasmine.hassan1@nhs.net (J.H.); kaushiki.bakaya@nhs.net (K.B.); 2 Department of Neurosurgery, University College London Hospitals NHS Foundation Trust, London WC1N 3BG, UK; austin.koh@ucl.ac.uk (C.H.K.); joan.grieve@nhs.net (J.G.); h.marcus@ucl.ac.uk (H.J.M.); 3 Lorn and Islands Hospital, Oban PA34 4HH, UK; gordon.caldwell2@nhs.scot; 4 Centre for Obesity & Metabolism, Department of Experimental & Translational Medicine, Division of Medicine, University College London, London WC1E 6BT, UK

**Keywords:** clinical consultations, COVID-19 pandemic, quality, telemedicine

## Abstract

The coronavirus disease 2019 pandemic may have affected the quality of clinical consultations. The objective was to use 10 proposed quality indicator questions to assess outpatient consultation quality; to assess whether the recent shift to telemedicine during the pandemic has affected consultation quality; and to determine whether consultation quality is associated with satisfaction and consultation outcome. A cross-sectional study was used to survey clinicians and patients after outpatient consultations (1 February to 31 March 2021). The consultation quality score (CQS) was the sum of ‘yes’ responses to the survey questions. In total, 78% (538/690) of consultations conducted were assessed by a patient, clinician, or both. Patient survey response rate was 60% (415/690) and clinician 42% (291/690). Face-to-face consultations had a greater CQS than telephone (patients and clinicians < 0.001). A greater CQS was associated with higher overall satisfaction (clinicians log-odds: 0.77 ± 0.52, *p* = 0.004; patients log-odds: 1.35 ± 0.57, *p* < 0.001) and with definitive consultation outcomes (clinician log-odds: 0.44 ± 0.36, *p* = 0.03). In conclusion, consultation quality is assessable; the shift to telemedicine has negatively impacted consultation quality; and high-quality consultations are associated with greater satisfaction and definitive consultation outcome decisions.

## 1. Introduction

**Figure 1:**
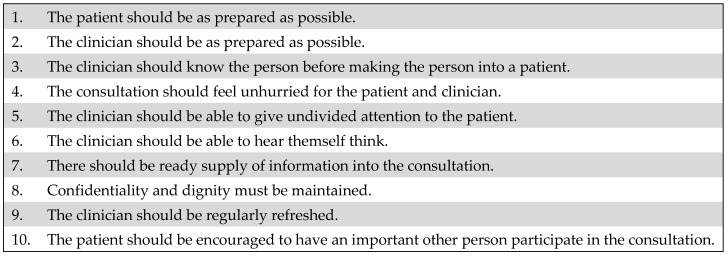
Ten quality indicators for clinical consultation [3,4].

Clinical consultation skills are a core component of medical student training and postgraduate practice, and essential in the delivery of high-quality healthcare [1,2]. Some clinicians, however, feel the standard of modern-day clinical consultations is poor and have been advocating for improvement [3]. Dr Gordon Caldwell has proposed 10 indicators to improve the quality of clinical consultations, and thereby improve patient outcomes, patient experience, patient safety, and staff satisfaction (see Figure 1) [4].

The diversion of health care resources required to manage the virus throughout the COVID-19 pandemic has caused delays in the routine care of patients with chronic medical conditions [5]. Many health services rapidly adopted methods to conduct remote consultations to reduce the viral spread [6,7]. It has been suggested that the impact of the COVID-19 pandemic on both the frequency and method of clinical consultations has had an additional deleterious effect on the quality of clinical consultations.

The aims of our work were: firstly, to use these proposed indicators to assess the quality of consultations in outpatient clinics; secondly, to determine whether the shift toward telephone consultations throughout the COVID-19 pandemic has impacted the quality of consultations; and thirdly, to assess whether the quality of consultation was associated with patient satisfaction, clinician satisfaction, and outcome of the consultation.

## 2. Materials and Methods

We adopted a cross-sectional study design and the Strengthening the Reporting of Observational Studies in Epidemiology (STROBE) Statement in the preparation of this manuscript.

### 2.1. Settings and Participants

This study was conducted at UCLH, a tertiary referral service. Participants were patients assessed in medical (endocrinology) and surgical (neurosurgery) outpatient clinics between the 1 February 2021 and the 31 March 2021, as well as the clinicians who reviewed them. The consultations were conducted by clinicians at a consultant or specialist registrar level under supervision.

### 2.2. Variables and Data Sources

Clinicians and patients were *both* surveyed after each outpatient appointment (see File S1 in Supplementary Materials). The survey questions generated were derived from the previously reported 10 indicators to improve the quality of clinical consultations (see Figure 1). In doing so, we took a consensus-based pragmatic approach balancing the rich detail of the clinical consultation with what we thought we could feasibly measure and analyze. The clinicians autonomously completed a survey immediately after each consultation either via paper copy or electronically. The clinicians were reminded via email at the beginning of each week to complete the surveys following clinical consultations. Patients were identified from clinic lists on electronic health records and were surveyed via telephone and called within one week of their consultation. A second call was attempted within 24 h if patients failed to answer initially. All patients were surveyed by one of three medical students (who had never met the patients).

In each survey, demographic details of the patient (age and sex) and the type of consultation (face-to-face vs. telephone, initial vs. review appointment) were recorded. Participants were asked the outcome of the consultation (further investigations, monitoring, treatment, or discharged). Participants were then asked a series of questions, aiming to assess different aspects of the quality of the clinical consultation. The sum of the number of ‘yes’ responses to the quality indicator survey questions provided a consultation quality score (CQS). This was a score out of 9 for patients and out of 10 for clinicians, given an extra clinician question about feeling refreshed and having the opportunity for adequate breaks.

Finally, clinicians and patients were asked if overall they were satisfied with the consultation and if they had any further comments to share. The patient group was also asked two extra follow up questions. Patients were asked if they would like the option of a telephone consultation after the COVID-19 pandemic ends. If the patient reported a negative response to question 10, i.e., that their relative or friend had not been invited to participate in the consultation, they were asked if they would have liked this to have occurred.

### 2.3. Study Size and Statistical Methods

No formal power calculation was performed. Instead, the sample size was determined by a pragmatic approach, with a minimum of 500 consultation assessments thought to be meaningful for analysis and an achievable target over the two-month study period.

Survey data was recorded using Microsoft Excel on a secure hospital server and fully anonymized datasets were generated and exported for further analysis in line with our institutional information governance framework. Statistical analysis was conducted using R statistical programming [8]. Graphs were plotted using the package ggplot2 [9]. Dichotomous dependent variables were analyzed using logistic regressions. Continuous dependent variables with binary independent variables were analyzed using a two-tailed t-test with Welch’s correction. The threshold for statistical significance was set at α < 0.05. Adjustments for multiple comparisons were made at the level of each analysis, using Benjamini–Hochberg’s method to control for false discovery rates [10]. The data is presented as mean and 95% confidence intervals.

## 3. Results

Over the two-month study period 14 clinicians conducted 690 consultations.

Survey response:

Of the 690 consultations conducted, 538 were assessed using either a patient survey, a clinician survey, or both (538/690, 78%). Overall, 706 surveys were completed. Patient surveys were completed after 415 of the consultations (415/690, a survey response rate of 60%) and clinicians surveys were completed after 291 of the consultations (291/690, a survey response rate of 42%). There were 168 consultations that were assessed by both a clinician and a patient survey. No surveys were partially completed.

Of the 275 patients phoned who did not complete a survey (275/690, 40%), 184 did not answer the phone call, 52 declined to participate, 27 were unable to complete the phone survey due to a language barrier, or 12 for another reason. The 399 consultations that were not assessed by clinicians (399/690, 58%) were the result of surveys not being returned to the researchers.

Survey results:

The patients attending the consultations had a mean age of 49 years (range 16–88 years) and 60% were female (322/538). The details of the consultation setting, type, method, and outcome are outlined in Table 1. Most consultations were in the setting of a medical clinic (444/538, 83%), and were review appointments (483/538, 90%). More telephone consultations (370/538, 69%) were conducted, compared to face-to-face consultations (168/538, 31%). Regarding the outcome of the consultation, a decision was made (i.e., treatment or discharge) after 12% (67/538) of the consultations whilst the decision was pending (i.e., further investigations or monitoring) after 87% (471/538) of consultations.

### 3.1. Quality of Consultation

The number of yes vs. no responses to the quality indicator survey questions for both the clinician and patient surveys are outlined in Table 2. For the majority of survey questions (Question [Q] 1–2, 4–6, and 8), the response was ‘yes’ greater than 90% of the time. Clinicians were refreshed and took adequate breaks (Q9) 87% of the time. Enquiry about a patient’s occupation or interests was variable (Q3—answered yes on the clinician survey (C) 70%, answered yes on the patient survey (*p*) 49%). Clinicians reported that the information needed for the consultation was frequently missing (Q7—C 47%, *p* 90%) and relatives or friends were most often not invited to participate (Q10—C 23%, *p* 10%). Of the 372 patients surveyed who had not been offered the option of a family or friend attending the consultation, 28 (28/372, 8%) reported that they would have liked this to have occurred.

For the 168 consultations that were assessed by both the clinician and patient, the number of surveys with a difference in the yes vs. no response to the quality indicator survey questions is outlined in Table 3. Patients reported more frequently that clinicians did not take the time to ask about hobbies or interests (clinician and patient survey discordance 30%). Clinicians reported more frequently that the required information was missing (clinician and patient survey discordance 52%).

### 3.2. Quality of Consultation and Consultation Method

Both patients and clinicians assessed face-to-face consultations to be of a significantly higher overall quality (indicated by a higher CQS) than telephone consultations (*p* < 0.001 for both clinicians and patients). Both were significantly more likely to feel that occupation was elicited during face-to-face consultations (*p* = 0.004 for both clinicians and patients). Patients were more likely to report that a relative or friend was invited to be present during face-to-face consultations (*p* = 0.03), although clinicians reported no significant difference (*p* = 0.77). Despite the higher satisfaction with face-to-face consultations, 73% of patients reported that they would like to have the option of a telephone a consultation after the pandemic ends (303/415, 73%).

### 3.3. Quality of Consultation and Satisfaction

Both clinicians (285/291) and patients (406/415) reported 98% satisfaction with the consultations (see Table 2). For the consultations assessed by both parties (n = 168), there was only 2% discordance between clinicians and patients regarding overall consultation satisfaction (3/168 patients reported overall dissatisfaction with the consultation, whilst 168/168 clinicians reported overall satisfaction) (see Table 3).

A greater clinician-assessed CQS was significantly associated with greater likelihood of clinician satisfaction (log-odds: 0.77 ± 0.52; *p* = 0.004; *n* = 291). A greater patient-assessed CQS was significantly associated with a greater likelihood of patient satisfaction (log-odds: 1.35 ± 0.57; *p* < 0.001; *n* = 415).

In a subset of 168 consultations assessed by both the patient and the clinician, we found no association between clinician-assessed CQS and patient satisfaction (*p* = 0.75). We could not assess whether patient-assessed CQS had any impact on clinician satisfaction, as no clinicians indicated dissatisfaction in this subset.

The best predictors of patient satisfaction are outlined in Table 4. By univariable analysis, these were: an unhurried consultation (Q4, log-odds: 2.99 ± 1.52, *p* < 0.001), knowledge of the reason for the consultation (Q1, log-odds: 2.80 ± 1.50; *p* = 0.001), clinicians knowing the details of their case (Q2, log-odds: 2.38 ± 1.46, *p* = 0.004), and all the required information being available for the consultation (Q7, log-odds: 2.05 ± 1.36, *p* = 0.006). In multivariable logistic regression, an unhurried consultation (Q4, log-odds: 2.83 ± 1.86, *p* = 0.003), knowledge of the reason for the consultation (Q1, log-odds: 1.93 ± 1.73, *p* = 0.03), and the required information being available for the consultation (Q7 2.41 ± 1.56, *p* = 0.002), were also found to be significant predictors of patient satisfaction.

The best predictors of clinician satisfaction are also outlined in Table 4. By univariable analysis, these were: knowledge of the reason for consultation (Q1, log-odds: 4.25 ± 1.88, *p* < 0.001), the consultation being distraction free (Q6, log-odds: 2.76 ± 1.67, *p* = 0.005), an unhurried consultation (Q4, log-odds: 2.27 ± 1.78, *p* = 0.04), and confidentially and dignity being maintained (Q8, log-odds:2.64 ± 2.36, *p* = 0.06). In multivariable logistic regression, knowledge of the reason for the consultation (Q1, log-odds: 4.08 ± 2.13, *p* = <0.001) and the consultation being distraction free (Q6, log-odds: 2.56 ± 2.03, *p* = 0.01), were also found to be significant predictors of clinician satisfaction.

### 3.4. Quality of Consultation and Consultation Outcome

The outcomes were dichotomized into discharged or treated (decision made) versus further investigation or monitoring (decision pending). There was a significant association between clinician assessed CQS and decision made (log-odds: 0.44 ± 0.36; *p* = 0.03). Of the individual components of the CQS, sufficient information was the sole significant predictor of treatment decision being made (log-odds: 1.69 ± 0.86; *p* = 0.002). There were no significant associations between overall patient assessed CQS or individual quality components, with decision outcome.

### 3.5. Other Survey Results

When comparing the setting of medical and surgical clinics, there was no significant difference in patient-assessed CQS (*p* = 0.94). The clinician-assessed CQS was greater in the surgical clinic setting (*p* = 0.01).

In the free comment section (answered by 35 patients), a common theme related to difficulties organizing blood tests (reported by 7 patients). Patients also requested to see the same clinician at each appointment (reported by 6 patients).

## 4. Discussion

### 4.1. Principal Findings

**Figure 2:**
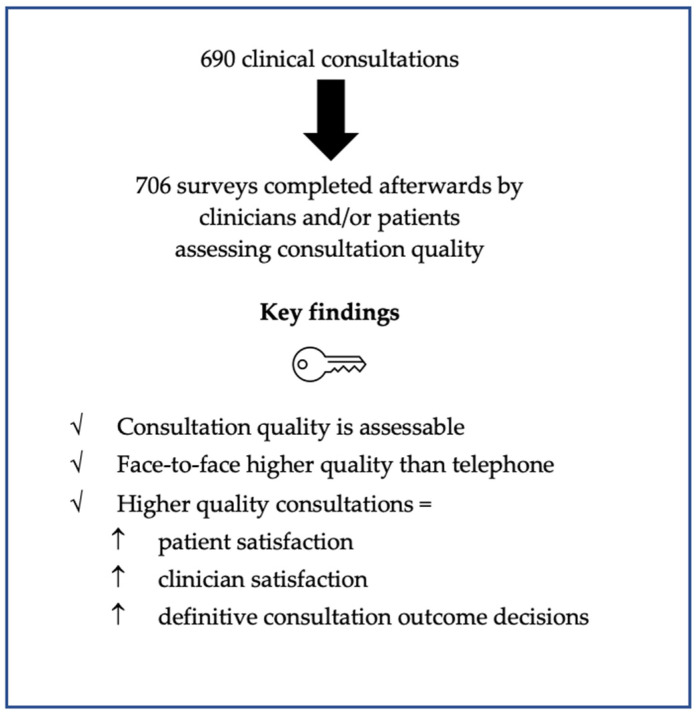
Key study findings.

This study demonstrated the following: firstly, it is possible to assess the quality of consultation; secondly that the shift from face-to-face to telephone clinic has impacted on this quality; and thirdly, that high quality consultations are associated with greater patient satisfaction, greater clinician satisfaction, and more definitive decisions (see Figure 2). This has important ramifications, suggesting quality of consultation is a measurable and important facet of patient care.

Overall, the quality of the consultations was high with a positive response reported for the majority of the clinical consultation quality indicators. Most patients knew why they were attending, the clinicians were prepared, and there was adequate time for the consultation. Consultations were generally free of interruptions or distractions and confidentially was maintained. Breaks were taken by most clinicians.

There were three quality indicators where improvement could be made. The clinicians often did not ask about the patient’s occupation or interests, reported by patients in approximately half of the consultations. In many of the consultations a friend or relative was not invited to participate. Clinicians reported in half of the consultations that they did not have the required information.

For both clinicians and patients, face-to-face consultations were felt to be of a higher overall quality than remote consultations. Despite this, most patients would like the option of a telephone consultation after the COVID-19 pandemic ends.

Both clinicians and patients reported high satisfaction with the clinical consultations. There was high concordance between clinician and patient overall consultation satisfaction when the same consultation was assessed by both parties. A higher consultation quality score (the overall sum of the positive responses to the quality indicator survey questions) was significantly associated with greater satisfaction with the consultation. For both clinicians and patients, patients knowing the reason for the consultation and an unhurried consultation were predictors of overall satisfaction. Other predictors for patient satisfaction were the clinician knowing the details of their case and the required information being available. Predictors of clinician satisfaction were the consultations being distraction free, where confidentially and dignity was maintained.

When the outcome of the consultation was a definitive decision (i.e., treatment or discharge), the clinicians rated the consultation to be of higher quality. The required information being available for the consultation was the sole predictor of a definitive decision being made regarding the outcome of the consultation.

### 4.2. Comparison to Other Studies

Previous work has aimed to develop tools to measure the quality of consultations, although differing aspects of the consultation were assessed [11,12]. Two thirds of consultations were conducted via telephone, aligning with the trend to increase delivery of digital health care going forward [13,14]. We found face-to-face consultations were of higher quality compared to telephone consultations, consistent with research conducted in primary care prior to the pandemic [15]. Despite this finding, overall high satisfaction was still reported, in keeping with other literature reporting high satisfaction with telephone consultations throughout the pandemic [16,17,18,19].

Improving the quality of consultations is more challenging when consulting remotely. Previous research has suggested less rapport building is undertaken during telephone consultations compared to face-to face [20] and loss of non-verbal communication is a challenge when consulting remotely [21]. Primary care professions interviewed about their experience consulting during the pandemic, reported feeling that remote consultations created emotional distance between themselves and patients [22]. Video consultations may be superior to telephone in enabling rapport to be built [23].

Eight percent of patients who were not offered the option of a support person would have liked this to have occurred, highlighting a missed opportunity. Literature has reported the beneficial role a companion can play in a clinical consultation [24,25]. Examining ways to involve family or friends in remote consultations is particularly important for older individuals, a group that may be less familiar with digital technology and who are thought to be disproportionately affected by the pandemic [26].

The required information was frequently missing. Delays to investigations have been a common occurrence throughout the pandemic [5] and increased difficulties obtaining pathology results when using telehealth has been reported [27]. Methods to ensure results are available efficiently and reliably when consulting remotely remains a key area of focus going forward. The availability of the necessary information was also associated with a definite decision being made regarding the consultation outcome. Maximizing efficiency is important given the long-lasting impact the COVID-19 pandemic is predicted to have on already stretched health care resources [28].

Reassuringly, clinicians reported having time to take adequate breaks, although improvement can be made in 13% of consultations. Previous studies have explored the link between clinician fatigue and altered decision making [29,30,31], of particular importance currently given increased clinician burnout induced by the pressures of the COVID-19 pandemic [32,33].

Pat McBride, head of patient and family services at The Pituitary Foundation, a patient support organization, provided a valuable insight from a patient perspective. One area not considered by the proposed consultation quality indicators is the frustration for patients when there is a delay for a consultation to start. The increased use of remote consulting may provide an opportunity to examine and implement methods to better address this issue, given patients no longer have to be physically waiting in a reception area.

### 4.3. Strengths and Limitations

A strength of this study was that multiple perspective and dimensions of the consultation (patient and clinician satisfaction, as well as consultation outcome) were examined. A large number of patients were surveyed across both medical and surgical clinics. To minimize recall bias, clinicians completed surveys on the day of the consultation and patients were surveyed within one week.

A limitation of this study was that 184 of patients identified did not complete the survey given they were uncontactable via phone after two attempts. A further 27 were unable to complete the phone survey due to a language barrier. It is possible that this group of patients may have reported more problems and lower satisfaction with telephone consultations. Clinicians completed the surveys after 42% of consultations. The reason clinician surveys were not returned to researchers is unknown. Unfortunately, we were not able to follow up missing clinician surveys as we had not routinely collected data identifying individual clinicians on the survey forms. It possible that clinicians were unable to complete surveys due to time pressure, meaning our survey failed to capture those who were unable to take adequate breaks. This is a single-center study at a large London referral hospital; thus, the findings of this survey may not be representative of other services, including smaller hospitals, who were more stretched for resources during the pandemic. Given this was an observational study, the stability of a patient’s condition may have influenced the choice of a face-to-face or remote consultation and therefore the findings.

## 5. Conclusions

We demonstrated that assessing the quality of clinical consultations is possible and established that the shift from face-to-face to telephone clinics during the COVID-19 pandemic has impacted on this quality. In addition, high quality consultations are associated with greater patient satisfaction, greater clinician satisfaction, and more definitive decisions being made regarding the consultation outcome. To further improve the quality of consultations, clinicians should make steps to better understand their patients as people, for example, knowing their occupations and interests and more consistently offering to involve a patient’s family or friends in their healthcare. Improved methods should be implemented to ensure that all relevant information is available at the time of the consultation. Consideration should be given to the patient group most suitable for telephone consultations, given the challenges of maintaining quality when consulting remotely. It is important that these areas of improvement are considered as we start to look towards a healthcare model that comprises increasing levels of remote consultations and deal with the backlog of the impact of the COVID-19 pandemic.

## Figures and Tables

**Table 1:** Details of the consultation setting, type, method, and outcome.

Consultation Details *n* = 538	
Setting	Medical clinic: 444 (83%) Surgical clinic: 94 (17%)
Type	Initial consultation: 55 (10%) Review consultation: 483 (90%)
Method	Face-to-face consultation: 168 (31%) Telephone consultation: 370 (69%)
Outcome	Decision pending: 471 (88%) Further investigations: 196 Monitoring: 275 Decision made: 67 (12%) Treatment: 46 Discharged: 21

**Table 2:** Clinician and patient survey results.

Question (Q) Number	Clinician Survey Question	*n* = 291	Patient Survey Question	*n* = 415
Q1	Did the patient know why they were attending the consultation?	yes: 284 (98%) no: 7 (2%)	Did you know why you were attending the consultation?	yes: 400 (96%) no: 15 (4%)
Q2	Did you have the opportunity to review the case before the consultation?	yes: 289 (99%) no: 2 (1%)	Did the clinician know the details of your case?	yes: 394 (95%) no: 21 (5%)
Q3	Did you ask and document the patient’s occupation and/or interests?	yes: 203 (70%) no: 88 (30%)	Did the clinician ask about your occupation and/or interests?	yes: 203 (49%) no: 212 (51%)
Q4	Did the consultation time feel adequate and unhurried?	yes: 275 (95%) no: 16 (5%)	Did the consultation time feel adequate and unhurried?	yes: 402 (97%) no: 13 (3%)
Q5	Was the consultation free of interruptions? E.g., being called by a colleague during the consultation.	yes: 274 (94%) no: 17 (6%) Of those who answered no: Staff interrupting: 11 IT issues: 2 Other: 4	Was the consultation free of interruptions? E.g., you or the clinician received other calls during the consultation.	yes: 409 (99%) no: 6 (1%) Of those who answered no: Staff interrupting: 1 IT issues: 1 Other: 4
Q6	Was the consultation free of distractions? E.g., nearby building works.	yes: 272 (93%) no: 19 (7%) Of those who answered no: Construction: 11 Other noise: 4 Patient at work: 2 Patient driving: 1 Other: 1	Was the consultation free of distractions? E.g., nearby building works.	yes: 412 (99%) no: 3 (1%) Of those who answered no: Construction: 1 Other noise: 2
Q7	Did you have all the information needed to conduct the consultation?	yes: 137 (47%) no: 154 (53%) Of those who answered no: Results: 150 * Scans: 8 Information: 3 Other: 2	Did the clinician have the information needed to conduct the consultation?	yes: 374 (90%) no: 41 (10%) Of those who answered no: Results: 19 Scans: 6 Information: 10 Other: 6
Q8	Were confidentiality and dignity maintained throughout the consultation? E.g., was the telephone consultation able to be conducted in a confidential setting?	yes: 286 (98%) no: 5 (2%) Of those who answered no: Patient outside home: 5	Were confidentiality and dignity maintained throughout the consultation? E.g., was the telephone consultation able to be conducted in a confidential setting?	yes: 414 (100%) no: 1 (<1%)
Q9	Were breaks taken as needed throughout the clinic and, if an afternoon clinic, did you have a lunch break?	yes: 253 (87%) no: 38 (13%)	No survey question	
Q10	Was the patient’s friend or relative invited to participate in the consultation? E.g., via speaker phone or sitting next to the patient throughout the consultation.	yes: 68 (23%) no: 223 (77%)	Was your friend or relative invited to participate in the consultation? E.g., via speaker phone or sitting next to you throughout the consultation.	yes: 43 (10%) no 372 (90%)
Q11	Overall, were you satisfied with the consultation?	yes: 285 (98%) no: 6 (2%)	Overall, were you satisfied with the consultation?	yes: 406 (98%) no: 9 (2%)

* 8 patients missing > 1 information sources.

**Table 3:** Differences between clinician and patient survey responses when assessing the same consultation.

Question (Q) Number	Clinician Survey Question *n* = 168	Patient Survey Question *n* = 168	Number of Surveys with a Difference in y/*n* Survey Response for Patient and Clinician Assessing Same Consultation *n* = 168
Q1	Did the patient know why they were attending the consultation?	Did you know why you were attending the consultation?	8 (5%)
Q2	Did you have the opportunity to review the case before the consultation?	Did the clinician know the details of your case?	9 (5%)
Q3	Did you ask and document the patient’s occupation and/or interests?	Did the clinician ask about your occupation and/or interests?	47 (30%)
Q4	Did the consultation time feel adequate and unhurried?	Did the consultation time feel adequate and unhurried?	11 (7%)
Q5	Was the consultation free of interruptions? E.g., being called by a colleague during the consultation.	Was the consultation free of interruptions? E.g., you or the clinician received other calls during the consultation.	13 (8%)
Q6	Was the consultation free of distractions? E.g., nearby building works.	Was the consultation free of distractions? E.g., nearby building works.	9 (5%)
Q7	Did you have all the information needed to conduct the consultation?	Did the clinician have the information needed to conduct the consultation?	88 (52%)
Q8	Were confidentiality and dignity maintained throughout the consultation? E.g., was the telephone consultation able to be conducted in a confidential setting?	Were confidentiality and dignity maintained throughout the consultation? E.g., was the telephone consultation able to be conducted in a confidential setting?	3 (2%)
Q10	Was the patient’s friend or relative invited to participate in the consultation? E.g., via speaker phone or sitting next to the patient throughout the consultation.	Was your friend or relative invited to participate in the consultation? E.g., via speaker phone or sitting next to you throughout the consultation.	18 (11%)
Q11	Overall, were you satisfied with the consultation?	Overall, were you satisfied with the consultation?	3 (2%)

**Table 4:** Predictors of clinician and patient satisfaction.

	Univariate Logistic Regression Analysis				Multivariate Logistic Regression Analysis			
	Clinician		Patient		Clinician		Patient	
Survey Question (Q)	Log-Odds	*p*	Log-Odds	*p*	Log-Odds	*p*	Log-Odds	*p*
Q1	4.25 ± 1.88	<0.001	2.80 ± 1.50	0.001	4.08 ± 2.13	<0.001	1.93 ± 1.73	0.03
Q2	na *	na *	2.38 ± 1.46	0.004				
Q3	0.13 ± 1.72	0.99	2.07 ± 2.09	0.08				
Q4	2.27 ± 1.78	0.04	2.99 ± 1.52	<0.001			2.83 ± 1.86	0.003
Q5	1.21 ± 2.21	0.42	−13.77± 3165.60	>0.99				
Q6	2.76 ± 1.67	0.005	−12.76 ± 2715.34	>0.99	2.56 ± 2.03	0.01		
Q7	1.52 ± 2.16	0.30	2.05 ± 1.36	0.006			2.41 ± 1.56	0.002
Q8	2.64 ± 2.36	0.06	na *	na *				
Q9	−15.85 ± 3419.28	>0.99	No survey question					
Q10	−0.55 ± 1.72	0.69	−0.08 ± 2.10	>0.99				

* variable not analyzed as ≤2 people in the group agreed or disagreed.

## Data Availability

Datasets generated and analyzed during the study are available from the corresponding author on reasonable request.

## Supplementary Materials

The following supporting information can be downloaded at: https://www.mdpi.com/article/10.3390/clinpract12040058/s1, File S1: Clinician and patient surveys.
