# Supplementary material for: Quality in Clinical Consultations: A Cross-Sectional Study
Source: Clin Pract. 2022 Jul 14;12(4):545–56. doi: 10.3390/clinpract12040058 (PMC9326638; doi:10.3390/clinpract12040058)
Supplement: Supplementary file 1 [file clinpract-12-00058-s001.zip › clinpract-1762483-File S1.pdf]

Clinician Survey: *Quality Indicators for Clinical Consultations*

Consultation date	_ _ / _ _ / _ _ _ _
Type of consultation	☐ Face to face ☐ Telephone
Patient MRN	
Patient age	
Patient sex	
Consultation type	☐ Initial appointment ☐ Review
Consultation outcome	☐ Further investigations ☐ Treatment ☐ Monitoring ☐ Discharged

Did the patient know why they were attending the consultation?	☐ Yes	☐ No	
Did you have the opportunity to review the case before the consultation?	☐ Yes	☐ No	
Did you ask and document the patient’s occupation and/or interests?	☐ Yes	☐ No	
Did the consultation time feel adequate and unhurried?	☐ Yes	☐ No	
Was the consultation free of interruptions? (eg: being called by a colleague during the consultation)	☐ Yes	☐ No	If no, please specify:
Was the consultation free of distractions? (eg: nearby building works)	☐ Yes	☐ No	If no, please specify:
Did you have all the information needed to conduct the consultation?	☐ Yes	☐ No	If no, please specify:<br>☐ Lab results ☐ Imaging results<br>☐ Information from GP or specialists<br>☐ Other:<br>______________________________________<br>______
Were confidentiality and dignity maintained throughout the consultation? (eg: was the telephone consultation able to be conducted in a confidential setting?)	☐ Yes	☐ No	If no, please specify:
Were breaks taken as needed throughout the clinic and, if an afternoon clinic, did you have a lunch break?	☐ Yes	☐ No	
Was the patient’s friend or relative invited to participate in the consultation? (eg: via speaker phone or sitting next to the patient throughout the consultation)	☐ Yes	☐ No	
Overall, were you satisfied with the consultation?	☐ Yes	☐ No	
Any further comments?			

Patient Survey: *Quality Indicators for Clinical Consultations*

Consultation date	_ _ / _ _ / _ _ _ _
Type of consultation	☐ Face to face ☐ Telephone
Patient MRN	
Patient age	
Patient sex	☐ Male ☐ Female
Consultation type	☐ Initial appointment ☐ Review
Consultation outcome	☐ Further investigations ☐ Treatment ☐ Monitoring ☐ Discharged

Did you know why you were attending the consultation?	☐ Yes	☐ No	
Did the clinician know the details of your case?	☐ Yes	☐ No	
Did the clinician ask about your occupation and/or interests?	☐ Yes	☐ No	
Did the consultation time feel adequate and unhurried?	☐ Yes	☐ No	
Was the consultation free of interruptions? (eg: you or the clinician received other calls during the consultation)	☐ Yes	☐ No	If no, please specify:
Was the consultation free of distractions? (eg: nearby building works)	☐ Yes	☐ No	If no, please specify:
Did the clinician have the information needed to conduct the consultation?	☐ Yes	☐ No	If no, please specify:<br>☐ Lab results ☐ Imaging results<br>☐ Information from GP or specialists<br>☐ Other: ______________________________
Were confidentiality and dignity maintained throughout the consultation? (eg: was the telephone consultation able to be conducted in a confidential setting?)	☐ Yes	☐ No	If no, please specify:
Was your friend or relative invited to participate in the consultation? (eg: via speaker phone or sitting next to you throughout the consultation)	☐ Yes	☐ No	If no, would you have liked this to have occurred? ☐ Yes ☐ No
Overall, were you satisfied with the consultation?	☐ Yes	☐ No	
After the pandemic ends, would you like the option of having your consultation via the telephone?	☐ Yes	☐ No	
Any further comments?	☐ Yes	☐ No	
